# Gender Differences in Remission and Recovery of Schizophrenic and Schizoaffective Patients: Preliminary Results of a Prospective Cohort Study

**DOI:** 10.1155/2012/576369

**Published:** 2012-01-16

**Authors:** Bernardo Carpiniello, Federica Pinna, Massimo Tusconi, Enrico Zaccheddu, Francesca Fatteri

**Affiliations:** Section of Psychiatry, Department of Public Health, University of Cagliari, Liguria Street 13, 09127 Cagliari, Italy

## Abstract

The aim of the paper was to evaluate rates of clinical remission and recovery according to gender in a cohort of chronic outpatients attending a university community mental health center who had been diagnosed with schizophrenia and schizoaffective disorder according to DSM-IV-TR. A sample of 100 consecutive outpatients (70 males and 30 females) underwent comprehensive psychiatric evaluation using the Structured Clinical Interview for Diagnosis of Axis I and II DSM-IV (SCID-I and SCID-II, Version R) and an assessment of psychopathology, social functioning, clinical severity, subjective wellbeing, and quality of life, respectively by means of PANSS (Positive and Negative Syndrome Scale), PSP (Personal and Social Performance), CGI-SCH (Clinical Global Impression—Schizophrenia scale), SWN-S (Subjective Well-being under Neuroleptics—scale), and WHOQOL (WHO Quality of Life). Rates of clinical remission and recovery according to different criteria were calculated by gender. Higher rates of clinical remission and recovery were generally observed in females than males, a result consistent with literature data. Overall findings from the paper support the hypothesis of a better outcome of the disorders in women, even in the very long term.

## 1. Introduction

The importance of gender differences in psychiatry is widely acknowledged, given its relevance for a better understanding of biological and psychosocial risk factors, time course, outcome, and response to treatments of major mental disorders, in particular schizophrenia and psychotic spectrum disorders [1–4]. Moreover, gender differences may play a crucial role in the early diagnosis and treatment of schizophrenia [5–7]. Indeed, a significant body of data revealed gender differences in terms of incidence rates, neurobiological factors, familial transmission, age of onset, clinical features, course and outcome, treatment response, compliance, and tolerability of drug treatments [2, 3, 8, 9]. According to the majority of authors, males manifest the disease earlier [2, 10, 11] and display more severe premorbid dysfunctions, intellectual impairment, and social deficits [10, 12–15], although data present in the literature are somewhat conflicting at times [5]. However, course and outcome of the illness [2, 10, 16], as well as the first episode of psychosis itself, are usually more severe and disabling in males [17–19]. On the contrary, women manifest onset symptoms later in life [2, 10, 11, 17], complete their studies, get married, or establish intimate relationships more frequently than males, showing less frequently negative [6, 7, 12, 13, 20–22] and affective [6, 10, 23] symptoms. Furthermore, women show a lower tendency towards substance abuse and antisocial behavior [24–27], better response to treatment [10, 28], higher rate of compliance, but an increased vulnerability to side effects of drugs [29]. A higher age of onset, better premorbid functioning, and a more favorable course of the disease may justify the better clinical and social outcome generally observed in women [10]. Notwithstanding the large amount of data relating to clinical and psychosocial outcome in schizophrenia and related disorders, only a limited number of studies to date have evaluated clinical outcome using operational criteria for clinical and functional remission [30, 31]. Moreover, social outcome has prevalently been assessed by means of social-demographic parameters (marital status, occupation, and level of autonomy) or other nonspecific measures [31]. As a consequence, the existence of gender differences with regard to rates of clinical or psychosocial remission and recovery remains a matter of debate in the light of the conflicting evidence reported [31–39]. These contrasting results are partly due to relevant methodological differences, particularly criteria used in the definition of either remission or recovery. Taking into account the limited number of studies conducted on psychotic patients in community settings and evaluated by means of operationalized criteria, the present study aimed to evaluate rates of clinical remission and recovery according to gender in a cohort of chronic outpatients with a diagnosis of schizophrenia and schizoaffective disorder who were attending a community mental health center.

## 2. Methods

### 2.1. Sample

In the context of a prospective follow-up study, all patients with a diagnosis of schizophrenia or schizoaffective disorder according to DSM-IV-TR attending a university community mental health centre between 1 January and 31 December 2010 were enrolled consecutively. Patients with other comorbid disorders were also included in the study, although those affected by comorbid mental retardation or organic brain diseases were excluded. A sample of 100 consecutive outpatients (70 males, mean age 42.49 ± 7.75 yrs; 30 females; mean age 44.70 ± 11.76 yrs) who met the above-mentioned inclusion/exclusion criteria was studied. All patients were submitted to standard care provided in community mental health centers in Italy (pharmacological treatment, clinical monitoring at least on a monthly basis, home care when required, and psychosocial and rehabilitation interventions tailored to patient's needs).

### 2.2. Ratings

All patients underwent comprehensive psychiatric evaluation by means of the Structured Clinical Interview for Diagnosis of Axis I DSM-IV (SCID-I Research Version) [40] and for Axis II of DSM-IV (SCID-II) [41], after having signed an informed consent form. Interviews were conducted by residents in psychiatry trained in the use of the instruments by a specialist; interrater reliability, assessed using Cohen's K before the study, was higher than 0.80.

Personal and social data, and clinical history were collected on the basis of a structured interview purpose-developed for the present study. Severity of symptoms was evaluated by means of PANSS (Positive and Negative Syndrome Scale) [42] and Clinical Global Impression—Schizophrenia scale (CGI-SCH) [43]; functioning, subjective wellbeing and quality of life were, respectively, evaluated by means of PSP (Personal and Social Performance) [44], Subjective Wellbeing under Neuroleptics (SWN-S) [45], and WHO Quality of Life Brief questionnaire (WHOQOL-Brief) [46].

PANSS (Positive and Negative Syndrome Scale) consists of 30 items grouped into 3 distinct clusters (positive symptoms, negative symptoms, general psychopathological symptoms). The manual accompanying the scale provides a detailed explanation of individual items and criteria of quantification of symptoms that are rated on a 7-point scale.

PSP (Personal and Social Performance Scale), developed from SOFAS (Social Occupational Functioning Scale), assesses social functioning of patients with schizophrenia on 4 main areas: social activities, personal and social relationships, self-care and disturbing/aggressive behaviors. The PSP must be completed on the basis of data provided by the patient, family, or staff-member in charge of the patient. For each area a score ranging from 0 (no disability) to 5 (very severe disability) is attributed according to specific criteria. A comprehensive overall score ranging from 1 (maximum dysfunction) to 100 (maximum functioning) is attributed, based on score obtained at each single area.

CGI-SCH is the adapted version of the CGI (Clinical Global Impression Rating Scale) [43], one of the main rating scales currently used in the comprehensive assessment of psychopathology. The CGI scale comprises 3 main scores: severity of illness, global improvement, and efficacy index. The CGI-SCH, as adapted for use in schizophrenia, provides for the assessment of severity and improvement of positive, negative, cognitive, symptoms and depression over the week before the visit.

SWN (Subjective Wellbeing under Neuroleptic Treatment) is a self-administered rating scale developed by Naber in 1995, aimed at evaluating the psychological and physical wellbeing of patients treated with neuroleptics. For the purpose of the study the short version (20 items, including 5 subscales: mental functions, self-control, physical function, emotional control, and social capability) was used.

The WHOQOL (World Health Organization Quality of Life Scale) is a self-evaluated questionnaire developed by the World Health Organization (WHO QOL Group) to assess subjective quality of life. In the present study the 26-item short version (WHOQOL-BRIEF) was used, allowing us to obtain four subscores focusing on the quality of life in 4 areas, respectively (physical, psychological, social relationships, and environment).

### 2.3. Criteria for Clinical Remission and Recovery

To evaluate clinical remission, criteria developed by the “Remission of Schizophrenia Working Group” [30] based on ratings at 8 focal symptoms in positive, negative, and general psychopathology subscales of PANSS (P1, P2, P3, N1, N4, N6, G5, G9) were applied. The patient is judged to be in clinical remission when scores obtained at each of these items are less than or equal to 3 over a six-month period. Due to the fact that baseline data from an ongoing, prospective follow-up study was used, as suggested by the Remission of Schizophrenia Working Group severity was adopted in evaluating clinical remission, whilst duration was not taken into account. Moreover, to define a state of clinical remission [47], a CGI-SCH score equal or less than 3 was required. Given that the criteria published by Andreasen for clinical remission are based on eight selected items from PANSS, thus excluding all items which significantly contribute towards overall clinical picture and quality of life, such as depressive and other symptoms [48], we decided to apply other more restrictive criteria. For the purpose of this study therefore, clinical remission was defined as (a) the meeting of both Andreasen's and CGI-SCH criteria; (2) achieving a score equal or less than 3 at each item of the Positive and Negative Scale of PANSS (extended PANSS criterion); (3) obtaining a score equal to or less than 3 at all items of PANSS (overall PANSS criterion).

In view of the lack of consensus in literature as to the definition and criteria of recovery [49], we adopted specific criteria purpose-developed for the present study. Recovery was primarily defined as the simultaneous fulfillment of Andreasen's criteria for clinical remission together with the presence of a “functional remission” as evaluated by PSP (score equal or greater to 70) and a “subjective remission,” in terms of a full subjective wellbeing according to SWN scale (score equal or greater than 80). Secondary criteria for recovery were defined as (1) clinical remission plus functional remission and (2) clinical remission plus subjective remission. Criteria for clinical remission and recovery are summarized in Table 1.

### 2.4. Statistical Analysis

Categorical data were analyzed using Pearson's *χ* ^2^ test (chi-square) or Fisher's exact test; continuous variables were assessed by means of Student's *t*-test for independent samples. Data analyses were performed using SPSS 19.0. Level of significance was set at a *P* value ≤0.05 for two-tailed hypothesis

## 3. Results

### 3.1. Sociodemographic Characteristics and Clinical History according to Gender

Significant gender differences were detected with regard to marital status. No other differences in sociodemographic characteristics were found (Table 2).

Females were more frequently married (*χ* ^2^ = 13.280, *df* = 2, *P* = 0.001) and with children than males (*χ* ^2^ = 7.563, *df* = 1, *P* = 0.012). The age at onset of psychosis was higher in women (26.10 ± 12.26 years) than in men (21.61 ± 7.67 years) although the difference did not achieve statistical significance (*t* = − 1.854, *df* = 39.082, *P* = 0.071). Age at first treatment for psychotic disorder was higher in women (30.90 ± 12.23 yrs) than in men (24.93 ± 8.04 yrs; *t* = −2.455, *df* = 40.172, *P* = 0.018). No gender differences were found in mean number of hospitalizations (1.29 ± 1.185 in men and 1.63 ± 1.732 in women; *t* = −0.977, *df* = 41.458, *P* = 0.334). However, men were characterized by a higher number of months since the last hospitalization (124.13 ± 108.52) than women (71.84 ± 66.43) (*t* = 2.392, *df* = 53.325, *P* = 0.020). No significant differences were revealed in duration of untreated psychosis (DUP), considered as average time in months between the onset of psychotic symptoms and the first treatment: 20.90 ± 40.27 mo. in men versus 44.55 ± 91.17 mo. in women (*t* = −1.342, *df* = 32.757, *P* = 0.189); the type of course (episodic with complete remission, episodic with residual symptoms or continuous; Table 3); percentage of patients having undergone at least one hospitalization in their lifetime (69.6%, *n* = 48 males and 63.3%, *n* = 19 females; *χ* ^2^ = 0.371, *df* = 1, *P* = 0.641) and who had attempted suicide or manifested self-harming behaviors (27.5%, *n* = 19 males and 37.9%, *n* = 11 females; *χ* ^2^ = 1.039, *df* = 1, *P* = 0.342). The proportion of patients who had committed crimes was higher in males (11.6%, *n* = 8) than females (0%; *χ* ^2^ = 3.661, *df* = 1, *P* = 0.053).

### 3.2. Axis I and II Diagnosis according to Gender

On the basis of data obtained at SCID-I interviews, a higher frequency of schizophrenia was found among males (M = 48.8%; F = 26.7%) while a more frequent diagnosis of schizoaffective disorder was detected among females (M = 51.4%; F = 73.3%; *P* = 0.042).

No significant gender differences were observed in Axis I comorbidity: comorbid anxiety disorders were diagnosed in 21.5% males and 20% females; other disorders were found in 5.7% males and 6.7% females. At SCID II 22.9% of males and 30.0% of females were diagnosed as having a personality disorder, although the difference was not statistically significant. Moreover, no relevant differences among genders were found with regard to personality clusters, with the sole exception of personality disorder not otherwise specified, more frequently represented in females (23.3%) than males (8.6%; *P* = 0.056).

### 3.3. Treatment Prescribed

The large majority of patients were on psychopharmacological treatment without any significant difference according to gender (males = 100%; females = 96.6%). Similarly, no difference was found in type of drug treatment (typical or atypical antipsychotics, antidepressants, mood stabilizers, and anticholinergics), with the exception of benzodiazepines, prescribed somewhat more frequently to males (64.3%) than females (43.3%; *P* = 0.052).

More women (23.3%) than men (7.2%) underwent individual psychotherapeutic treatment (*P* = 0.024) while no significant differences were detected with regard to group therapies and family interventions. 18.8% of males and 30.0% of females were undergoing rehabilitation treatment, the difference not being statistically significant; however, female patients were more frequently involved in expressive therapies (art therapy; 23.3%) than males (7.2%; *P* = 0.024).

### 3.4. Psychopathological Severity according to Gender

No significant differences were found among genders in mean scores achieved at PANSS total scale, general psychopathology scale, and positive and negative scales. Moreover, no significant gender differences were detected in mean scores on the overall severity scale and each one of the subscales (positive symptoms, negative symptoms, depressive symptoms, and cognitive deficits) of CGI-SCH. When focusing on individual items of PANSS, significant gender differences were found only for items N7 (stereotyped thinking; males = 1.60 ± 1.027, females = 1.23 ± 0.679, *P* < 0.05); G3 (feelings of guilt; males = 1.37 ± 0.871; females = 1.90 ± 1.348, *P* < 0.05), and G5 (postural mannerisms; males = 1.09 ± 0.329; females = 1.00 ± 0.000, *P* < 0.05).

### 3.5. Social Functioning according to Gender

Males displayed significantly higher mean scores than females on PSP “disturbing and aggressive behaviors” (males = 0.38 ± 0.75; females = 0.13 ± 0.434; *P* = 0.046). With regard to PSP total score, a greater dysfunction was detected in males (46.07 ± 26.449) than in females (33.93 ± 31.858), although this difference was not significant from a statistical point of view (*P* = 0.073). Taking into account patients with a PSP score equal to or higher than 70, 10% of males and 30% of females were considered “functionally remitted” in view of their adequate functioning, a difference devoid of statistical significance.

### 3.6. Subjective Wellbeing according to Gender

Women showed significantly higher scores (18.66 ± 3.243) than males (16.81 ± 4.044) at the “self-control” subscale of SWN (*P* = 0.020). Considering patients with an SWN total score equal to or higher than 80, no significant gender differences were found. 65.7% of males and 70% of females were considered in “subjective remission” (true subjective wellbeing).

### 3.7. Quality of Life according to Gender

No significant gender differences were revealed in subjective quality of life for the four areas considered (physical, psychological, social relationships, and environment) in the WHOQOL-BREF questionnaire.

### 3.8. Clinical Remission according to Gender

Data obtained according to the different criteria of clinical remission adopted are reported in Table 4. A lower percentage of male (48.6%, *n* = 34) than female patients (60.0%, *n* = 18) were deemed in clinical remission according to the criteria of Andreasen, a finding devoid of statistical significance. Similarly, no significant gender differences emerged when CGI-SCH criteria were used to evaluate clinical remission (males = 56.7%, *n* = 38; females = 48.1%, *n* = 13). When more restrictive criteria were applied (Andreasen's and CGI-SCH criteria combined), once again no difference was detected between genders (41.8%  *n* = 28 males and 46.2%  *n* = 13 females). On applying more restrictive criteria to evaluate clinical remission (a score less than or equal to 3 for all items of the PANSS positive and negative scales), clinical remission was observed in 31.4% of males (*n* = 22) and 46.7% of females (*n* = 14), although devoid of statistical significance. Even following the use of extremely restrictive criteria to define clinical remission (scores less than or equal to 3 for all PANSS items), no difference was found between genders, given that 22.9% of males (*n* = 16) and 26.7% of females (*n* = 8) were seen to be in clinical remission using these criteria. 

### 3.9. Recovery in Relation to Gender

Patients who were clinically remitted as established by Andreasen's criteria were viewed as being “recovered,” as “adequately functioning” in line with PSP total score and in a state of subjective wellbeing on the basis of SWN total score (Table 5), with a significantly higher proportion of females (16.7%, *n* = 5) than males (2.9%, *n* = 2) among the “recovered” (*P* = 0.024). When basing criteria for “recovery” on clinical remission and “adequate” functioning alone, once again a significantly higher proportion of females (26.7%, *n* = 8) than males (5.7%, *n* = 4) seemed to have “recovered” (*P* = 0.006). Similarly, when considering “recovery” only in terms of clinical remission associated with a true subjective wellbeing, a higher albeit not significant proportion of females (41.4%, *n* = 12) than males (25.7%, *n* = 18) were “recovered”(*P* = 0.123). 

## 4. Discussion

The present study provides a cross-sectional picture of a cohort of typical chronic psychotic patients attending a community mental health service in Italy. The patient sample was made up largely of middle aged, low income subjects with a long psychiatric history who lived in the community. As expected, a number of gender differences were revealed in the sample described here. Sociodemographic conditions of women were somewhat better than those of males, as shown by the higher frequency of female patients who were married with children, a finding that is substantially congruent with data present in the literature [8], probably reflecting the later onset of schizophrenia, a better premorbid adjustment [10, 12, 13] and less severe symptoms at onset among females [17]. In the sample studied, a higher proportion of schizoaffective disorders was found among females, a finding partially congruent with literature data reporting a higher frequency of affective symptoms in women [6, 10, 11, 23]. Although statistical significance was not reached, likely due to the limited number of subjects investigated, the younger age at onset of the disorder and the consequent early start of treatment in males observed in this study is fully in agreement with previous findings [2, 10, 11]. The more frequent prevalence of male offenders found in our study, together with a higher rate of disturbing and aggressive behaviors as evaluated by means of PSP, tend to confirm data from the literature reporting a stronger association of violent crimes in mental disorders with the male gender [49, 50]. No substantial gender differences were detected with regard to duration of untreated psychosis, a result which is partly congruent with literature data reporting on first-onset psychoses [51], and in contrast with others showing a longer DUI among males [52]. In accordance with literature findings (2) no gender differences were detected in the sample studied in symptom course pattern, although there was a considerable lack of literature studies reporting on clinical course according to DSM-IV criteria.

The absence of gender differences in current pharmacological treatment may indicate that, in spite of a series of literature reports demonstrating substantial differences between the genders in terms of response to and tolerability of treatments, in clinical practice males and females are treated in a comparable manner, thus reflecting the lack of gender-specific treatment guidelines [4]. In this study, female patients more frequently underwent individual psychotherapy, a finding which might be interpreted as the consequence of a higher propensity compared to males to resort to psychological interventions rather than to actual differences in therapeutic needs. Interestingly, no differences were detected in the frequency of rehabilitation activities between genders, with the sole exception of art therapies, which were more frequent among female patients. These findings are partly in contrast with literature data, underlining how women are less involved in rehabilitation activities, probably due to the lower degree of disability generally attributed to the female sex, and to lower needs of clinical and psychosocial care [53, 54]. Moreover, women are reportedly less involved in job placement or educational programs [8], although no significant gender differences were observed in the present sample with regard to these interventions. The data obtained in this study showed substantially similar levels and quality of psychopathology, as revealed by PANSS and CGI-SCH, in males and females; these findings are in contrast with data present in the literature, generally reporting a higher incidence of negative symptoms in males, and affective symptoms in females [6, 23, 31]. However, in the present study the more frequent observation of schizoaffective disorders in women may reflect a higher frequency of mood symptoms in females throughout the longitudinal course of the illness.

Following application of Andreasen's et al. [30] criteria, higher rates of clinical remission were detected for women than for men, although differences were not statistically significant; likewise, similar results emerged when other criteria were used. However, as expected rates of remission progressively decreased when more restrictive sets of criteria were considered. Moreover, a higher albeit not significant percentage of women showed an adequate functioning and were considered in “functional remission”. Furthermore, compared to males, women more frequently report a condition of subjective wellbeing on SWN scale, with statistically significant differences in the “self-control” subscale. Recent studies utilizing operational criteria to define clinical and functional remission show contrasting results. A study by Galderisi et al. [31] failed to find a statistically significant difference in rates of clinical remission between genders and higher rates of functional remission in females, a difference at the limits of statistical significance; Brugnoli et al. [55] found a higher frequency of clinical remission among females, while no difference between the sexes was found by Karow et al. [56].

When taking into account the main criteria adopted in the present study, that is, a state of clinical remission together with an adequate functioning and a true subjective wellbeing, “recovery” was significantly more frequent among female patients; similar results were obtained when recovery was only based on clinical remission and functional status. These results are fundamentally congruent with evidence from the literature. Indeed, in a longitudinal prospective study lasting 20 years Grossman et al. [32] revealed a trend for a better overall outcome and higher rates of recovery among women with schizophrenia and other psychotic disorders, thus disconfirming the hypothesis advanced by some authors that in women with schizophrenia outcome worsened over time, largely resembling that observed in men [33–35]. The SOHO study [30] reported a higher frequency of full recovery among females, presenting a lower global severity, less negative symptoms, and better social functioning at baseline. Similarly, Albert et al. [37] found that recovery was predicted, among other factors, by female sex.

Prior to the drawing of any conclusion from the data collected in the present study, a series of limitations should be taken into account, including the cross-sectional nature of the study, the limited number of cases included, the exclusion of cases of mental retardation and organic brain disorders, the exclusion of duration criteria in defining clinical remission. On the other hand, the use of structured interviews to define diagnoses and of standardized methods in the evaluation of clinical and psychosocial variables, thus allowing remission and recovery to be assessed in a reliable manner should be considered strengths of the study. The overall data provided by this study of a cohort of chronic outpatients who were highly representative of typical psychotic patients attending community mental health centers in Italy, tend to confirm a best prognosis and lower overall severity of schizophrenic spectrum disorders in women than in men [2, 10, 14]. In particular, our data appear to demonstrate a better outcome not only in the short and middle term, but likewise in the long term in schizophrenic and schizoaffective women. This improved outcome is likely the result of both an intrinsic, less severe nature of the disorder and to a series of other positive factors related to treatment (i.e., better response, higher compliance) and psychosocial environment (i.e., higher social support) among women.

## Figures and Tables

**Table 1:** Clinical remission and recovery criteria.

	Psychopathology	Functioning	Subjective wellbeing
Clinical remission criteria			
Andreasen's criteria	Score ≤3 in items P1, P2, P3, N1, N4, N6, G5, G9 of PANSS		
CGI-SCH criteria	Overall score ≤3 at the CGI-SCH		
Andreasen's criteria + CGI-SCH criteria	Score ≤3 in items P1, P2, P3, N1, N4, N6, G5, G9 of PANSS, overall score ≤3 at the CGI-SCH		
Extended PANSS criteria	Score ≤3 at each one item of the Positive and Negative Scale of PANSS		
Overall PANSS criteria	Score ≤3 at all the items of PANSS		

Recovery criteria			
Clinical + functional + subjective wellbeing remission	Score ≤3 in items P1, P2, P3, N1, N4, N6, G5, G9 of PANSS	Score ≥70 at PSP Scale	Score ≥80 at SWN Scale
Clinical + functional remission	Score ≤3 in items P1, P2, P3, N1, N4, N6, G5, G9 of PANSS	Score ≥70 at PSP Scale	
Clinical + subjective wellbeing remission	Score ≤3 in items P1, P2, P3, N1, N4, N6, G5, G9 of PANSS		Score ≥80 at SWN Scale

**Table 2:** Sociodemographic characteristics of the sample according to gender.

	Males	Females	Statistics		*df*	*P* value
			*t* value	*χ* ^2^ test		
Age (mean years ± sd)	42.49 ± 7.751	44.70 ± 11.765	−0.947		98	0,350
Education (mean years ± sd)	10.37 ± 3.800	12.00 ± 4.235	−1.897		98	0,061
Marital status				13.280	2	0,001
Married (*N*, %)	5 (7.1)	6 (20.0)				
Separated (*N*, %)	1 (1.4)	5 (16.7)				
Widowed (*N*, %)	—	—				
Single (*N*, %)	64 (91.4)	19 (63, 3)				
Employment status				8.914	5	0,113
Employed	17 (24.33)	4 (13, 3)				
Housewives	—	2 (6.7)				
Students	2 (2.9)	2 (6.7)				
Retired	9 (12.9)	7 (23.3)				
Disability pension	32 (45.7)	10 (33.3)				
Unemployed	10 (14.3)	5 (16.7)				

**Table 3:** Course of illness according to gender.

Course of illness	Males	Females	*χ* ^2^ test	*df*	*P* value
Episodic with full remissions (*n*, %)	11 (16.2)	7 (23.3)	3.990	3	0.262
Episodic with residual symptoms (*n*, %)	14 (20.6)	3 (10.0)
Continuous (*n*, %)	39 (57.4)	20 (66.7)
Undefined (*n*, %)	4 (5.9)	—

**Table 4:** Clinical remission rates according to gender.

Criteria	Male (*n*, %)	Female (*n*, %)	*χ* ^2^ test	*df*	*P* value
Andreasen's criteria	34 (48.6)	18 (60.0)	1.099	1	0.383
CGI-SCH total score	38 (56.7)	13 (48.1)	0.569	1	0.498
CGI-SCH and Andreasen's criteria	28 (41.8)	12 (46.2)	0.145	1	0.816
PANSS (positive and negative items)	22 (31.4)	14 (46.7)	2.116	1	0.175
PANSS (all items)	16 (22.9)	8 (26.7)	0.167	1	0.799

**Table 5:** Recovery rates according to gender.

Recovery criteria	Male (*n*, %)	Female (*n*, %)	*χ* ^2^ test	*df*	*P* value
Clinical* + functional + subjective wellbeing remission	2 (2.9)	5 (16.7)	6.152	1	0.024
Clinical* + functional remission	4 (5.7)	8 (26.7)	8.730	1	0.006
Clinical* + subjective wellBeing remission	18 (25.7)	12 (41.4)	2.382	1	0.151

*Andreasen's et al. criteria [30].
